# The safety and feasibility of laparoscopic common bile duct exploration for treatment patients with previous abdominal surgery

**DOI:** 10.1038/s41598-017-15782-y

**Published:** 2017-11-13

**Authors:** Yong Huang, Qian Feng, Kai Wang, Xiaoli Xiong, Shubing Zou

**Affiliations:** 1 Department of General Surgery, The Second Affiliated Hospital of Nanchang University, Nanchang, 330006 China; 2 Department of Emergency and Critical Care Medicine, The Second Affiliated Hospital of Nanchang University, Nanchang, 330006 China

## Abstract

The purpose of this study was to evaluate the safety and feasibility of laparoscopic common bile duct exploration (LCBDE) in patients with previous abdominal surgery (PAS). The outcomes were compared in 139 patients (103 upper and 36 lower abdominal surgeries) with PAS and 361 without PAS who underwent LCBDE. The operative time, hospital stay, rate of open conversion, postoperative complications, duct clearance, and blood loss were compared. Patients with PAS had longer operative times (*P* = 0.006), higher hospital costs (*P* = 0.043), and a higher incidence of wound complications (*P* = 0.011) than those without PAS. However, there were no statistically significant in the open conversion rate, blood loss, hospital stay, bile leakage, biliary strictures, residual stones, and mortality between patients with and without PAS (*P* > 0.05). Moreover, compared with those without PAS, patients with previous upper abdominal surgery (PUAS) had longer operative times (*P* = 0.005), higher hospital costs (*P* = 0.030), and a higher open conversion rate (*P* = 0.043), but patients with previous lower abdominal surgery (PLAS) had a higher incidence of wound complications (*P* *=* 0.022). LCBDE is considered safe and feasible for patients with PAS, including those with PUAS.

## Introduction

Biliary stones have a high recurrence rate^1,2^. Many patients undergo reoperation or multiple operations for biliary stones. In the past, conventional open surgery for biliary stones in patients with previous abdominal surgery (PAS) had been the only effective treatment. However, with advances in techniques, laparoscopic common bile duct exploration (LCBDE) has proven to be safe, cost-effective, and reliable, regardless of whether it is performed as an elective or emergency procedure^3–6^. As a result, surgeons increasingly attempt LCBDE in patients with PAS, but few reports on the laparoscopic approach have been published.

In LCBDE for patients with PAS, the main obstacle is presumed technical difficulty associated with the presence of adhesions^1,7–9^. First, there is increased risk of injuring organs adherent to the abdominal wall during trocar insertion. Second, in addition to difficulty obtaining adequate exposure of the operating field, manual palpation cannot be performed in patients with previous upper abdominal surgery. Third, fibrotic adhesions can hinder the visualization and dissection of the perihepatic ligament, hepatoduodenal ligament, and hilar area, which can be an additional obstacle to an already challenging procedure. Moreover, these may increase the risk of intraoperative bleeding and injury of vascular or biliary structures. Therefore, there are few reports on LCBDE in patients with PAS. With advances in laparoscopic techniques, hepatectomy, cholecystectomy, appendectomy, colectomy, and gastrectomy have been safely performed in patients with PAS^10–13^. Hence, the hesitation to undertake LCBDE in patients with PAS should diminish.

Even though patients with PAS are good prospects for LCBDE, few studies on the impact of PAS on LCBDE have been reported. Furthermore, no clear guidelines are available for use of LCBDE in patients with PAS. The aim of this study was to compare the benefits and drawbacks of LCBDE in patients with and without PAS.

## Material and Methods

### Patients and grouping

This retrospective clinical study was conducted at the Department of General Surgery and approved by institutional review board (IRB) committee at the Second Affiliated Hospital of Nanchang University. Informed consent was obtained from all subjects. This study was carried out in accordance with established national and institutional ethical guidelines regarding the involvement of human subjects and the use of human tissues for research. All the patients underwent LCBDE (Table 1) were enrolled between January 2014 and July 2016 from the database, and were divided into two groups: 121 patients (Table 2) with PAS (open conversion *n* = 18) and 327 without PAS (open conversion *n* = 34). The two groups were further divided into those with T-tube drainage or primary closure. Residual stones were determined by T-tube cholangiography or abdominal computed tomography (CT) at 1–3 months postoperatively. Wound complication included wound infection and bleeding. The study was approved by the Second Affiliated Hospital of Nanchang University Ethics Committee, and specimens were taken with the patient’s full consent.

**Table 1:** Characteristics of patients with and without previous abdominal surgery.

	GroupA (*n* = 327)	GroupB (*n* = 121)	*P*
Gender (M/F)	144/183	53/68	0.989
Age (years)	56.6 ± 16.5	57.9 ± 14.8	0.045
Primary closure/T tube	235/90	81/40	
CBD size (mm)	12.9 ± 3.8	13.4 ± 4.6	0.238
Laboratory data			
WBC (10^9^/L)	10.6 ± 3.7	11.1 ± 3.9	0.258
AST (U/L)	54.3 (35.7,83.4)	51.2 (36.7,81.4)	0.788
ALT (U/L)	63.4 (39.5,108.5)	58.5 (35.7,85.6)	0.197
GGT (U/L)	194 (91,365)	192 (90,346)	0.831
Blood loss (ml)	25 (20,30)	26 (22,36)	0.297
Operative time (min)	177.1 ± 60.5	195.5 ± 64.6	**0**.**006**
Hospital stay (days)	15.0 ± 5.6	14.7 ± 5.0	0.636
Hospital expenses (WanRMB)	3.75 ± 1.23	4.04 ± 2.38	**0**.**043**
Conversion	34	18	0.246
Postoperative complication	31	16	0.251
Biliary stricture	2	2	0.296
Wound complication	4	7	**0**.**011**
Bile leakage	6	0	0.197
Residual stones	19	6	0.820
Mortality	0	1	0.270

GroupA, without previous abdominal surgery; GroupB, with previous abdominal surgery; WBC, blood cell count; AST, aspartate aminotransferase; ALT, alanine aminotransferase; GGT, gamma-glutamyltransferase; WanRMB, ten thousand renminbi.

**Table 2:** Types of previous abdominal operations.

Incision	Surgical history	*n*
Upper abdominal	CBD exploration	15
	Gastrectomy	14
	Splenectomy	1
	Hepatectomy	3
	Bowel resection	6
	Cholecystectomy	47
Lower abdominal	Appendectomy	11
	Hysterectomy	6
	Cesarotomy	18

### Inclusion criteria

1. No intrahepatic bile duct stricture identified before surgery; 2. all patients diagnosed using abdominal ultrasound, CT, endoscopic retrograde cholangiopancreatography, or magnetic resonance cholangiopancreatography; 3. diameter of common bile duct (CBD) ≥ 8 mm; 4. biliary tract malformation and tumor were excluded; and 5. no cardiopulmonary disorder that would contraindicate surgery.

### LCBDE procedure

Patients were placed supine and in lateral position at working height. Under general anesthesia, we used three-trocar or four-trocar techniques for the working port and choledochoscope. First, an infraumbilical trocar was inserted when the patient had no previous operative scar at the umbilicus. When the patient had an operative scar at the umbilicus, the first trocar site should be as far away as possible from the original surgical incision; if necessary, pneumoperitoneum should be established using the Hasson technique. After carbon dioxide pneumoperitoneum was created at 12 mmHg, a 30° oblique laparoscope was placed. Then, right-side 5-mm ports were placed for a coagulation hook or ultrasonic knife using blunt or sharp separation of abdominal adhesions. When the hepatoduodenal ligament was completely exposed, the CBD was carefully assessed; the anterior duct wall was incised using electrocoagulation, followed by placement of a 12# ventricular drainage tube. After saline lavage, the stones were removed with a choledochoscope and basket. After the stones were removed, a T tube was placed or the CBD underwent primary closure with barbed wire suture.

### Statistical Analysis

Statistical analysis was performed with SPSS software, version 17.0 (SPSS Inc., Chicago, IL, USA). Continuous variables were expressed as mean ± SD or median (range), and categorical variables were expressed as numbers. Continuous variables were compared using Student’s *t*-test test or the Mann-Whitney *U*-test, and categorical variables were compared using the *χ* ^2^ test or Fisher’s exact test. *P* < 0.05 was considered statistically significant.

## Results

### Patient characteristics

The patients who underwent LCBDE and were divided into two groups: 139 patients with PAS (103 with previous upper abdominal surgery [PUAS], 36 with previous lower abdominal surgery [PLAS], Table 3; open conversion *n* = 18) and 396 patients without PAS (open conversion *n* = 34). Patient demographic and clinical/laboratory data are presented in Table 1. There were no significant between-group differences in sex, age, and liver function (*P* > 0.05).

**Table 3:** Characteristics of patients with previous upper and lower abdominal surgery.

	Upper (*n* = 86)	*P* ^1^	Lower (*n* = 35)	*P* ^2^
Gender (M/F)	44/42	0.218	9/26	**0**.**040**
Age (years)	59.8 ± 14.3	0.100	53.2 ± 15.0	0.236
CBD size (mm)	14.1 ± 4.8	**0**.**019**	11.8 ± 3.4	0.098
Laboratory data				
WBC (10^9^/L)	11.0 ± 3.8	0.378	11.2 ± 4.2	0.378
AST (U/L)	49.3 (37.4,76.4)	0.305	46.9 (37.4,76.4)	0.305
ALT (U/L)	56.2 (35.7,82.3)	0.585	65.5 (38.2,83.2)	0.585
GGT (U/L)	187 (91.1,371.1)	0.786	186 (89,358)	0.786
Blood loss (ml)	26 (23,50)	0.430	25 (20,43)	0.430
Operative time (min)	194.8 ± 68.1	**0**.**005**	196.1 ± 55.3	0.075
Hospital stay (days)	14.7 ± 5.3	0.730	14.6 ± 4.5	0.697
Hospital expenses (WanRMB)	4.19 ± 2.75	**0**.**030**	3.67 ± 0.94	0.725
Conversion	17	**0**.**043**	1	0.348
Postoperative complication	11	0.366	4	0.762
Biliary stricture	1	0.505	0	1.000
Wound complication	4	0.062	3	**0**.**022**
Bile leakage	0	0.352	0	1.000
Residual stones	5	0.999	1	0.707
Mortality	1	0.208	0	—

^1^Statistically significant patients with previous upper abdominal surgery *vs* without previous abdominal surgery; ^2^Statistically significant patients with previous lower abdominal surgery *vs* without previous abdominal surgery; WBC, blood cell count; AST, aspartate aminotransferase; ALT, alanine aminotransferase; GGT, gamma-glutamyltransferase. WanRMB, ten thousand renminbi.

### Perioperative outcome

The surgical outcomes are shown in Table 1. The patients with PAS had longer operative times (*P* = 0.006), higher hospital costs (*P* = 0.043), and a higher incidence of wound complications (*P* = 0.011) than those without PAS. However, there were no significant differences in conversion rate, blood loss, hospital stay, bile leakage, biliary strictures, wound complications, residual stones, and mortality between the two groups (*P* > 0.05). One patient died of septic shock, but the association with the procedure was unclear. In addition, there were no significant differences in the white blood cell (WBC) count, aspartate aminotransferase (AST), alanine aminotransferase (ALT), and gamma-glutamyltransferase (GGT) (*P* > 0.05). These suggest that there was no difference in postoperative patient emergency status.

The 139 patients with PAS were divided into 103 with PUAS and 36 with PLAS. As shown in Table 3, patients with PUAS had longer operative times (*P* = 0.005), higher hospital costs (*P* = 0.030), and a higher open conversion rate (*P* = 0.043), but patients with PLAS had a higher incidence of wound complications (*P* = 0.022).

Patients were further grouped by primary closure or T-tube placement. In primary closure cases (Table 4), the blood loss (*P* = 0.031) of those with PUAS was more than in those without PUAS. However, in T-tube cases (Table 5), the mean operative time (233.9 ± 72.6 min) in those with PUAS was longer than that in those (195.2 ± 62.1 min) without PUAS (*P* = 0.007).

**Table 4:** Characteristics of primary closure patients.

	GroupA (*n* = 236)	GroupB (*n* = 58)	*P*
Gender (M/F)	101/135	29/28	0.372
Age (years)	56.5 ± 16.8	60.9 ± 13.3	0.064
CBD size (mm)	12.7 ± 3.7	14.1 ± 4.5	**0**.**011**
Laboratory data			
WBC (10^9^/L)	10.2 ± 3.6	10.8 ± 3.8	0.274
AST (U/L)	53.3 (35.5,84.0)	51.6 (39.7,76.9)	0.768
ALT (U/L)	61.8 (39.5,108.8)	52.1 (35.7,76.5)	0.147
GGT (U/L)	193 (81,370)	188 (104,370)	0.855
Blood loss (ml)	25 (20,30)	30 (24,75)	**0**.**031**
Operative time (min)	170.1 ± 58.5	175.9 ± 57.4	0.500
Hospital stay (days)	14.7 ± 4.9	14.7 ± 5.0	0.996
Hospital expenses (WanRMB)	3.64 ± 1.05	3.90 ± 1.42	0.117
Postoperative complication	31	6	0.566
Biliary stricture	1	1	0.356
Wound complication	2	2	0.176
Bile leakage	4	0	1.000
Residual stones	14	2	0.746
Mortality	0	1	0.197

GroupA, without previous abdominal surgery; GroupB, with previous abdominal surgery; WBC, blood cell count; AST, aspartate aminotransferase; ALT, alanine aminotransferase; GGT, gamma-glutamyltransferase. WanRMB, ten thousand renminbi.

**Table 5:** Characteristics and perioperative outcomes of T-tube patients.

	GroupA (*n* = 91)	GroupB (*n* = 28)	*P*
Gender (M:F)	43/48	15/14	0.974
Age (years)	56.9 ± 15.8	57.6 ± 16.3	0.845
CBD size (mm)	13.5 ± 3.8	13.9 ± 5.5	0.692
Laboratory data			
WBC (10^9^/L)	11.7 ± 3.9	11.5 ± 3.9	0.815
AST (U/L)	55.7 (36.0,82.6)	47.6 (33.7,74.5)	0.362
ALT (U/L)	66.1 (39.6,102.7)	65.5 (37.5,83.2)	0.395
GGT (U/L)	196 (115,350)	198 (89,394)	0.849
Blood loss (ml)	30 (23,35)	25 (20,43)	0.298
Operative time (min)	195.2 ± 62.1	233.9 ± 72.6	**0**.**007**
Hospital stay (days)	15.6 ± 7.0	14.8 ± 5.8	0.564
Hospital expenses (WanRMB)	4.03 ± 1.60	4.78 ± 4.36	0.175
Postoperative complication	11	8	**0**.**040**
Biliary stricture	1	0	1.000
Wound complication	3	5	**0**.**018**
Bile leakage	2	0	1.000
Residual stones	5	3	0.392

GroupA, without previous abdominal surgery; GroupB, with previous abdominal surgery; WBC, blood cell count; AST, aspartate aminotransferase; ALT, alanine aminotransferase; GGT, gamma-glutamyltransferase. WanRMB, ten thousand renminbi.

## Discussion

Conventional open surgery increases patient suffering, and also increases the economic burden. In addition, although endoscopic sphincterotomy (EST) can be used for treatment of primary and recurrent CBD stones^14^, it is associated with high complication and failure rates^15^. EST can lead to postoperative complications such as pancreatitis, perforation, blood loss, sepsis, and even death; EST can also cause disruption of the sphincter of Oddi, thus causing injury to the physiological barrier that prevents cholangitis due to duodenobiliary reflux^16,17^. Natsui *et al*.^18^ reported that the incidence of biliary bacterial contamination 30 months after EST was about 78%. Another study reported that the postoperative acute cholangitis rate was 2.4–10.3% for EST^19^. Because of the many drawbacks of EST and open surgery, LCBDE is readily accepted by the majority of patients because of the small surgical wound, less pain, rapid postoperative recovery, and fewer complications^4–6^. In the early laparoscopic era, PAS—especially biliary surgery—was considered a contraindication for laparoscopic surgery due to abdominal adhesions^20–22^. With rapid advances in laparoscopic equipment and technique, as well as the continued improvement in surgical skills, patients with PAS are no longer an absolute contraindication to laparoscopic surgery^10–13^. LCBDE has been performed in patients with PAS, with good clinical results, but few reports have been published.

This study was carryed to evaluate the safety and feasibility of LCBDE in patients with PAS. We found that patients with PAS had longer operative times and higher hospital costs than those without PAS. These results are understandable, because additional time is needed to dissect adhesions, and more instruments and medications are needed, increasing hospital costs^23^. We divided the patients with PAS into those with PUAS and those with PLAS. We found that patients with PUAS had longer operative times and higher hospital costs than those without PAS and PLAS; however, there was no difference between patients with PLAS and those without PAS. These results are in agreement with those of Karayiannakis *et al*.^11^, who reported that adhesions in patients with PUAS occurred more frequently, and were more extensive and denser than those in patients with PLAS; additionally, they found that the requirement for adhesiolysis performed by laproscopy was higher for patients with upper abdominal incisions than those with lower abdominal incisions. And the operating area of LCBDE is mainly concentrated in the upper abdominal. Therefore, it was not surprising for patients with PUAS to have higher open conversion rates than patients without PAS or patients with PLAS, as seen in our study. The high conversion rate was due to bowel injury and uncontrolled bleeding^23,24^, which helped obtain adequate exposure of the critical region of interest. Previous studies^25^ have also shown that the liver capsule and surgical wounds bleed easily during adhesion separation, and these factors may result in increased blood loss and a suboptimal operative field, increasing the risk of intraoperative complications. For biliary surgery, the incidence of biliary stricture, bile leakage, and residual stones were key indicators in the safety evaluation of biliary surgery^26^. Our results showed that there were no significant differences in the perioperative results between patients with and without PAS with respect to hospital stay, blood loss, and postoperative complications, which included bile leakage, biliary stricture, residual stones, and mortality; in fact, a higher complication rate was initially expected. This shows that LCBDE with PAS did not increase the amount of bleeding and postoperative complications. In addition, there were also no significant differences in the WBC, AST, ALT, and GGT levels. These indicate that there was no difference in postoperative patient emergency status. Therefore, there was no greater systemic effect with LCBDE in patients with PAS, LCBDE is safe and feasible for patients with PAS. As for the patients with PLAS had a higher incidence of wound complications than those without PAS or patients with PUAS. The possible reason for this is that the patients with PLAS were mainly female and subcutaneous fat is more abundant among females.

For LCBDE with PUAS, the most critical technical difficulty was the establishment of pneumoperitoneum and separation of abdominal adhesions^7–9,27^. Therefore, the approach to the abdomen must abide by the strict application of technical principles. The first trocar (umbilical observation port) site should be as far away as possible from the original surgical incision; if necessary, pneumoperitoneum should be established using the Hasson technique. That can effectively avoid bowel injury. Once the pneumoperitoneum is established, we can use direct vision and place the second trocar in the right anterior axillary line, where there are usually fewer adhesions. If the anatomy around the hilum has been destroyed by PAS, there is an increase in potential risks of surgery. Hence, it is particularly important to understand the anatomical rules of biliary surgery with PAS. The extent of intra-abdominal adhesions depends on the original incision, with adhesions being more extensive with an oblique incision under the right costal margin. We found that there were different degrees of adhesions: 1. adhesions between the liver diaphragm surface and abdominal wall; 2. the gastric antrum and omentum were adherent to the abdominal wall or were adherent to the right side of the round ligament of the liver; 3. the first and second parts of the duodenum shifted upward to block the porta hepatis; 4. the colon near the liver was moved up, leading to the disappearance of the lacunar space in the right inferior liver. Therefore, we try to separate the gastric antrum and omentum with an ultrasonic knife at the second trocar port in the right anterior axillary line, after which we can establish another port under direct vision. Then, we carefully dissect along the liver surface to restore the normal structure of the gastric pylorus, duodenum, and hepatoduodenal ligament. The duodenum with adhesions in the hilum is used as an anatomical “landmark”. The CBD is usually located deep in the duodenum; when the duodenum is dissected downward from the hilum, the CBD will be exposed. This method is called the “anterior approach to the hepatoduodenal ligament”. When the adhesions are difficult to dissect with this approach, we can dissect downward from the hepatic flexure along the right lateral side of the hepatoduodenal ligament to expose the right lower space of the liver and foramen of Winslow hole, to further reveal the right side of the CBD. In this way, we can determine the location of the CBD. This method is called the “right-side approach to the hepatoduodenal ligament”. If the CBD is difficult to identify, puncture with a scalpel or needle will confirm the CBD. If the gallbladder area cannot be accessed because of adhesions or severe bleeding, we switch to laparotomy.

Regarding the T-tube placement or primary closure of the CBD, there was no difference in the residual stone rate between the two groups in our study and even research demonstrate that primary duct closure after LCBDE is feasible and fewer complications than T-tube placement^28^, but the rate can reach up to 5%. Therefore, we believe that primary closure of the CBD must be performed with caution and must meet the following criteria: 1. CBD diameter > 8 mm, with few stones; 2. results of intraoperative exploration should coincide with those of preoperative evaluation; 3. stones should be completely removed, and intraoperative exploration should only find slight CBD wall inflammation and edema; 4. the CBD and duodenum are patent; 5. the surgeon is skilled at suture technique.

In conclusion, this study demonstrates that LCBDE for patients with a history of abdominal surgery is feasible and safe. Therefore, when LCBDE is planned for patients with a surgical history, the laparoscopic approach can be considered a good alternative.

## References

[1] Jan YY (1996). Surgical treatment of hepatolithiasis: long-term results. Surgery.

[2] Cheon YK, Cho YD, Moon JH, Lee JS, Shim CS (2009). Evaluation of long-term results and recurrent factors after operative and nonoperative treatment for hepatolithiasis. Surgery.

[3] Zhu B (2015). Early versus delayed laparoscopic common bile duct exploration for common bile duct stone-related nonsevere acute cholangitis. Sci Rep.

[4] Feng Qian (2016). Laparoscopic transcystic common bile duct exploration: Advantages over laparoscopic choledochotomy. PLoS ONE.

[5] Lei J (2016). Laparoscopic Transcystic Common Bile Duct Exploration: T-Shaped Incision of Cystic Duct with FREDDY Laser Lithotripsy. J Laparoendosc Adv Surg Tech A.

[6] Chan DS, Jain PA, Khalifa A, Hughes R, Baker AL (2014). Laparoscopic common bile duct exploration. Br J Surg.

[7] Cai LX (2016). Is Laparoscopic Hepatectomy a Safe, Feasible Procedure in Patients with a Previous Upper Abdominal Surgery?. Chin Med J (Engl).

[8] Yun KW (2012). Laparoscopic common bile duct exploration in patients with previous upper abdominal operations. Korean J Hepatobiliary Pancreat Surg.

[9] Wiebke EA (1996). Conversion of laparoscopic to open cholecystectomy. An analysis of risk factors. Surg Endosc.

[10] Tsunoda S (2014). Laparoscopic gastrectomy for patients with a history of upper abdominal surgery: results of a matched-pair analysis. Surg Today.

[11] Karayiannakis AJ, Polychronidis A, Perente S, Botaitis S, Simopoulos C (2004). Laparoscopic cholecystectomy in patients with previous upper or lower abdominal surgery. Surg Endosc.

[12] Law WL, Lee YM, Chu KW (2005). Previous abdominal operations do not affect the outcomes of laparoscopic colorectal surgery. Surg Endosc.

[13] Ahn KS, Han HS, Yoon YS, Cho JY, Kim JH (2011). Laparoscopic liver resection in patients with a history of upper abdominal surgery. World J Surg.

[14] Nishizawa T (2013). Effect of ursodeoxycholic acid and endoscopic sphincterotomy in long-term stenting for common bile duct stones. J Gastroenterol Hepatol.

[15] Freeman ML (1996). Complications of endoscopic biliary sphincterotomy. N Engl J Med.

[16] Wang P (2009). Risk factors for ERCP-related complications: a prospective multicenter study. Am J Gastroenterol.

[17] Freeman ML (1998). Complications of endoscopic sphincterotomy. Endoscopy.

[18] Natsui M, Honma T, Genda T, Nakadaira H (2011). Effects of endoscopic papillary balloon dilation and endoscopic sphincterotomy on bacterial contamination of the biliary tract. Eur J Gastroenterol Hepatol.

[19] Doi S (2013). Comparison of long-term outcomes after endoscopic sphincterotomy versus endoscopic papillary balloon dilation: a propensity score-based cohort analysis. J Gastroenterol.

[20] Tian J (2013). The safety and feasibility of reoperation for the treatment of hepatolithiasis by laparoscopic approach. Surg Endosc.

[21] Parsons JK, Jarrett TJ, Chow GK, Kavoussi LR (2002). The effect of previous abdominal surgery on urological laparoscopy. J Urol.

[22] Schirmer BD (1995). The impact of previous abdominal surgery on outcome following laparoscopic cholecystectomy. Surg Endosc.

[23] Beck DE (2000). Effect of previous surgery on abdominal opening time. Dis Colon Rectum.

[24] Hu M, Zhao G, Xu D, Liu R (2011). Laparoscopic repeat resection of recurrent hepatocellular carcinoma. World J Surg.

[25] Szomstein S (2006). Laparoscopic lysis of adhesions. World J Surg.

[26] Wu X (2012). Primary closure versus T-tube drainage in laparoscopic common bile duct exploration: a meta-analysis of randomized clinical trials. Langenbecks Arch Surg.

[27] Li LB, Cai XJ, Mou YP, Wei Q (2008). Reoperation of biliary tract by laparoscopy: experiences with 39 cases. World J Gastroenterol.

[28] Podda M (2016). Systematic review with meta-analysis of studies comparing primary duct closure and T-tube drainage after laparoscopic common bile duct exploration for choledocholithiasis. Surg Endosc.

